# Association of serum lipopolysaccharide-binding protein level with sensitization to food allergens in children

**DOI:** 10.1038/s41598-020-79241-x

**Published:** 2021-01-25

**Authors:** Eun Kyo Ha, Ju Hee Kim, Dong Keon Yon, Seung Won Lee, Mi Ae Kim, Kyung Suk Lee, Myongsoon Sung, Hye Mi Jee, Youn Ho Shin, Man Yong Han

**Affiliations:** 1 Department of Pediatris, Hallym University Kangnam Sacred Heart Hospital, Seoul, Republic of Korea; 2 Department of Pediatrics, CHA Bundang Medical Center, CHA University School of Medicine, CHA University, 351 Yatap-dong, Bundang-gu, Seongnam, Gyonggi-do 13496 Republic of Korea; 3 Department of Data Science, Sejong University College of Software Convergence, Seoul, Republic of Korea; 4 Department of Internal Medicine, CHA Bundang Medical Center, CHA University, Seongnam, Republic of Korea; 5 Department of Pediatrics, Hanyang University Guri Hospital, Hanyang University College of Medicine, Guri, Republic of Korea; 6 Department of Pediatrics, Soon Chun Hyang University Gumi Hospital, Soon Chun Hyang University College of Medicine, Gumi-si, Republic of Korea; 7 Deparment of Pediatrics, CHA Gangnam Medical Center, CHA University School of Medicine, CHA University, 566 Nonhyeon-ro, Gangnam-gu, Seoul, 06135 Republic of Korea; 8 Department of Pediatrics, Seoul National University Hospital, Seoul National University College of Medicine, Seoul, Republic of Korea

**Keywords:** Biomarkers, Medical research

## Abstract

Lipopolysaccharide (LPS)-binding protein (LBP) is an acute-phase reactant that mediates innate immune responses triggered by LPS. Recent studies indicated a positive correlation of circulating LBP level with chronic low-grade inflammation, a condition present in many non-communicable diseases. We determined the association of serum LBP concentration with allergic sensitization in a general pediatric population. Serum LBP was measured in a sample of children (n = 356; mean age = 9.6 ± 0.2 years) in this population-based cross-sectional study. Skin prick tests (SPTs) were performed to assess allergic sensitization to 22 common inhalant and food allergens. One hundred and seven children (30.1%) were nonsensitized, 160 (44.9%) were monosensitized, and 89 (25.0%) were polysensitized. Children who were mono- or polysensitized had a significantly higher median serum LBP level (25.5 ng/mL, inter-quartile range [IQR] 20.3–30.7) than those who were nonsensitized (20.3 ng/mL, IQR = 14.81–25.8, *P* < 0.0001). Multivariate logistic regression analysis with adjustment for confounders indicated that serum LBP level was positively associated with allergic sensitization overall (adjusted odds ratio [aOR] 1.041; 95% CI 1.007–1.076, *P* = 0.016), with sensitization to food allergens in particular (aOR 1.080, 95% CI 1.029–1.133, *P* = 0.002), but not with sensitization to aeroallergens (aOR 1.010, 95% CI 0.982–1.040, *P* = 0.467). LBP level was not associated with allergic diseases after adjustment. We suggest the possibility of sensitization to food allergens may be related to gut-derived low-grade inflammation, and large sized longitudinal investigations are needed to elucidate the relationship.

## Introduction

Sensitization by administration of a low level of lipopolysaccharides (LPS) from Gram-negative bacteria and a relevant allergen leads to TH2-type inflammation that is mediated by Toll-like receptor-4 (TLR-4)^1^. In contrast, intake of a high LPS level induces a TH1 response^1,2^, which corresponds well with the hygiene theory^3^. LPS is generally present at levels insufficient to stimulate macrophages in the absence of the accessory molecule, known as lipopolysaccharide-binding protein (LBP). Thus, LBP is a useful biomarker for activation of innate immune responses to microbial products^4^ and is widely used as a marker of LPS.

LBP is a 65-kDa soluble acute-phase protein mainly produced by hepatocytes^5^, intestinal epithelial cells^6^, and visceral adipocytes^7^. Recent studies demonstrated that serum LBP level correlates positively with obesity^8^, metabolic syndrome^9^, type 2 diabetes^10,11^, and atherosclerosis^12,13^. Thus, LBP may be used as a surrogate marker of “systemic chronic low-grade inflammation”, a condition present in many non-communicable diseases^14^.

Allergic diseases are also closely associated with systemic inflammation^15–18^, and childhood allergic sensitization is considered a prerequisite for the development of allergies^19,20^. Despite the possible links between systemic inflammation and allergic sensitization, few studies have investigated whether systemic low-grade inflammation accompanies childhood allergic sensitization^21^. Furthermore, no studies have determined whether LBP is associated with allergic sensitization and/or allergic diseases in humans.

We aimed to determine the association of serum LBP level with allergic sensitization and self-reported allergic diseases and to explore the possible mechanisms underlying this association.

## Results

The clinical characteristics of the 356 enrolled children are shown in Supplementary Table 1. A total of 160 children (44.9%) were monosensitized, 89 (25.0%) were polysensitized, and 107 (30.1%) were non-sensitized. There were 233 children (65.4%) sensitized to an aeroallergen such as house dust mites, 45 (12.6%) sensitized to plant food allergens, and 22 (6.2%) sensitized to class I food allergens; shrimp, port, mussel, egg, cod, and milk (Fig. E1).

### Allergic sensitization and LBP

**Figure 1:**
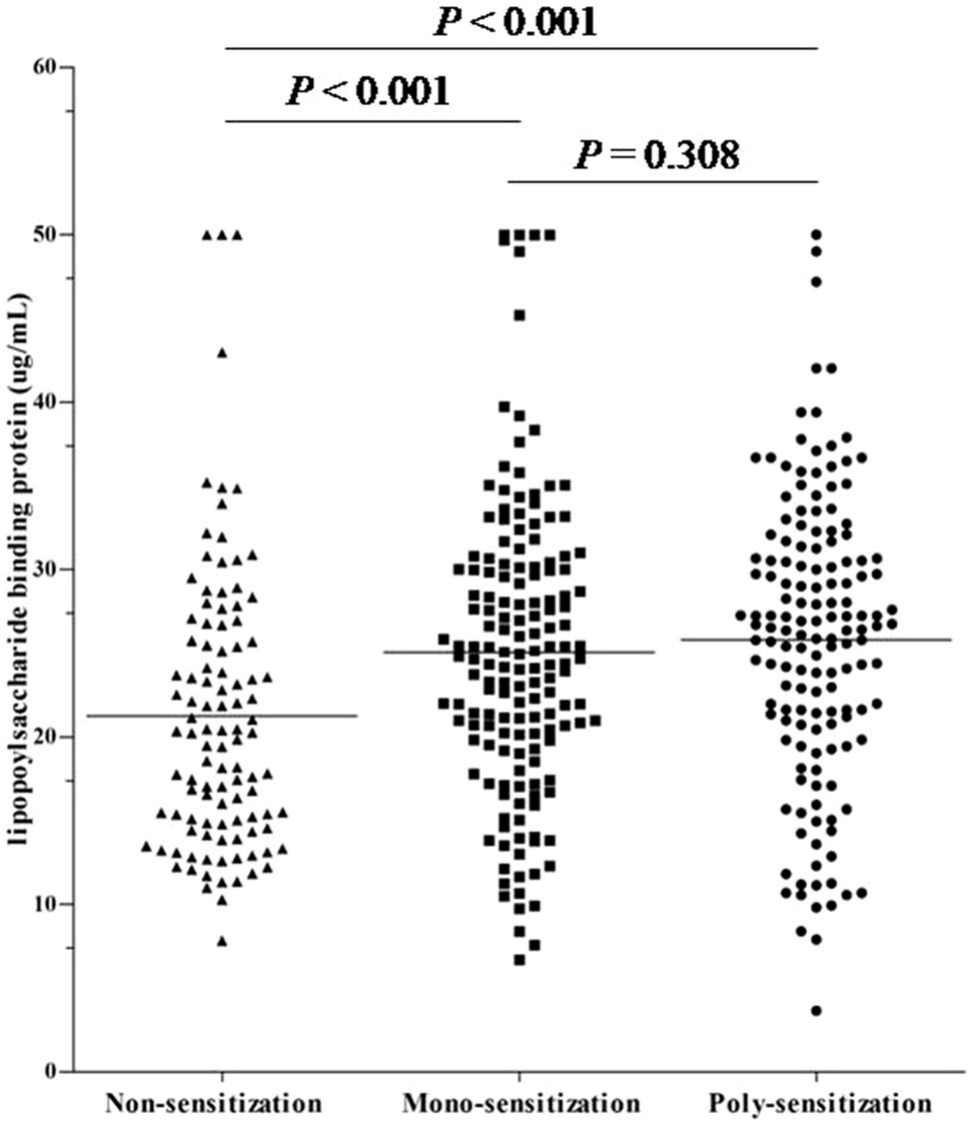
Serum LBP levels of children who were non-sensitized (n = 107), mono-sensitized (n = 160), and poly-sensitized (n = 89).

Analysis of serum LBP in the different groups indicated a greater median level in sensitized than nonsensitized children (25.5 ng/mL [IQR: 20.3–30.7] vs. 20.3 [IQR: 14.8–25.8], *P* < 0.001), but no significant difference in mono-sensitized and poly-sensitized children (*P* = 0.308, Fig. 1). LBP level was also positively associated with a higher risk of allergic sensitization (OR 1.052, 95% CI 1.020–1.085, *P* = 0.001), and this relationship remained significant after adjustment for confounders (adjusted odds ratio [aOR] 1.041, 95% CI 1.007–1.076, *P* = 0.016, Table 1). Children with an LBP level in the highest quartile had a significantly increased risk of allergic sensitization relative to those in the lowest quartile (aOR 3.599, 95% CI 1.576–8.222, *P* = 0.002, Table 1).

**Table 1:** Association of serum LBP level, as a continuous variable (top) and a categorical variable (bottom), with asthma, allergic rhinitis, atopic dermatitis, and allergic sensitization^*^.

LBP	Asthma		Allergic rhinitis		Atopic dermatitis		Allergic sensitization	
	OR (95% CI)	*P* value	OR (95% CI)	*P* value	OR (95% CI)	*P* value	OR (95% CI)	*P* value
**Continuous variable**								
Crude	1.013 (0.963–1.065)	0.615	1.027 (1.003–1.052)	**0.028**	0.971 (0.943–1.000)	**0.049**	1.052 (1.020–1.085)	**0.001**
Adjusted^†^	0.985 (0.922–1.052)	0.644	1.012 (0.982- 1.043)	0.438	0.984 (0.952–1.017)	0.350	1.037 (1.003–10.72)	**0.033**
Adjusted^‡^	0.974 (0.913–1.040)	0.435	1.012 (0.983–1.043)	0.416	0.993 (0.962–1.026)	0.691	1.040 (1.005–1.075)	**0.023**
Adjusted^§^	0.996 (0.931–1.066)	0.916	1.015 (0.985–1.045)	0.334	0.991 (0.959–1.024)	0.599	1.041 (1.007–1.076)	**0.016**
**Categorical variable**								
1 Q^†^	Ref		Ref		Ref		Ref	
2 Q	1.307 (0.173–9.845)	0.795	1.021 (0.466–2.238)	0.959	0.480 (0.199–1.155)	0.101	1.391 (0.665–2.910)	0.381
3 Q	1.749 (0.239–12.819)	0.582	1.715 (0.786–3.742)	0.176	0.556 (0.236–1.311)	0.180	2.744 (1.217–6.185)	**0.015**
4 Q	1.432 (0.191–10.760)	0.727	1.974 (0.917–4.246)	0.082	0.855 (0.384–1.906)	0.702	3.599 (1.576–8.222)	**0.002**

Allergic sensitization indicates one or more positive reactions (wheal diameter > 3 mm) to any allergen in the SPT.

*CI* confidence interval, *OR* odds ratio, *LBP* lipopolysaccharide-binding protein.

*Analyses are based on the 356 subjects using data on allergic sensitization (19 with asthma, 167 with allergic rhinitis, 80 with atopic dermatitis, and 249 with allergic sensitization).

OR was calculated using generalized linear regression with the logitfunction.

^†^Multivariate logistic regression adjusted for sex, age, body mass index z-score, parental asthma, allergic rhinitis, and atopic dermatitis, and household income (model I).

^‡^Multivariate logistic regression adjusted for sex, age, body mass index z-score, parental asthma, allergic rhinitis, and atopic dermatitis, and type of residency (model II).

^§^Multivariate logistic regression adjusted for sex, age, body mass index z-score, parental asthma, allergic rhinitis, and atopic dermatitis, and floor of residency (model III).

Children with self-reported allergic rhinitis and atopic dermatitis had a significantly higher median serum LBP level than controls (*P* = 0.028 and *P* = 0.049, respectively); however, this relationship did not remain significant after adjustment for sex, age, BMI z-score, parental allergic diseases, and household income (Model I), type of residency (Model II), or floor of residency (Model III) (Table 1). Children with asthma and controls had comparable LBP levels. There were no significant correlations of serum LBP level with levels of 25-hydroxyvitamin D_3_ or hemoglobin, or with counts of white blood cells, neutrophils, or total eosinophils (Suppl Table 2).

### Types of allergens and LBP

We also determined the relationship of sensitization to each type of allergen with LBP level (Table 2). Children with polysensitization had a significantly higher median serum LBP level than those with non-sensitization (OR 1.10, 95% CI 1.055–1.147, *P* < 0.001); this relationship remained significant after adjustment for confounders (aOR 1.110, 95% CI 1.059–1.164, *P* < 0.001). Of note, elevated LBP level was not associated with sensitization to aeroallergens including house dust mite (aOR 1.010, 95% CI 0.982–1.040, *P* = 0.467). However, LBP level was significantly associated with sensitization to any food allergen (aOR 1.080, 95% CI 1.029–1.133, *P* = 0.002), as well as sensitization to plant food allergens (aOR 1.256, *P* = 0.001) and sensitization to type I food allergens (aOR 1.128, *P* = 0.043).

**Table 2:** Association of serum LBP levelwith sensitization to specific allergens.

Dependent variable (allergen)	Lipopolysaccharide-binding protein					
	OR (95% CI)	*P* value	aOR (95% CI)*	*P* value	aOR (95% CI)^†^	*P* value
Non-sensitization^‡^	Ref		Ref		Ref	
Mono-sensitization	1.019 (0.985 to 1.055)	0.274	1.015 (0.979 to 1.052)	0.419	1.020 (0.986 to 1.056)	1.000
Poly-sensitization	1.100 (1.055 to 1.147)	** < 0.001**	1.110 (1.059 to 1.164)	** < 0.001**	1.105 (1.056 to 1.156)	**0.002**
**Allergens**						
Any inhalant allergens	1.017 (0.990 to 1.045)	0.227	1.010 (0.982 to 1.040)	0.467	1.017 (0.989 to 1.046)	1.000
Any food allergens	1.363 (1.156 to 1.607)	** < 0.001**	1.080 (1.029 to 1.133)	** < 0.001**	1.326 (1.206 to 1.457)	** < 0.001**

Allergic sensitization indicates one or more positive reactions (wheal diameter > 3 mm) to any allergen in the SPT.

*OR* odds ratio, *aOR* adjusted odds ratio, *LBP* lipopolysaccharide-binding protein.

*Multivariate logistic regression adjusted for sex (male), age, body mass index z-score, and household income.

^†^Multivariate logistic regression adjusted for sex (male), age, body mass index z-score, and household income. The Holm method was used to counteract the problem of multiple comparisons. ^‡^The *P* value for the association between LBP level and sensitization (non-, mono, and poly-sensitization) was from multinomial regression.

**Figure 2:**
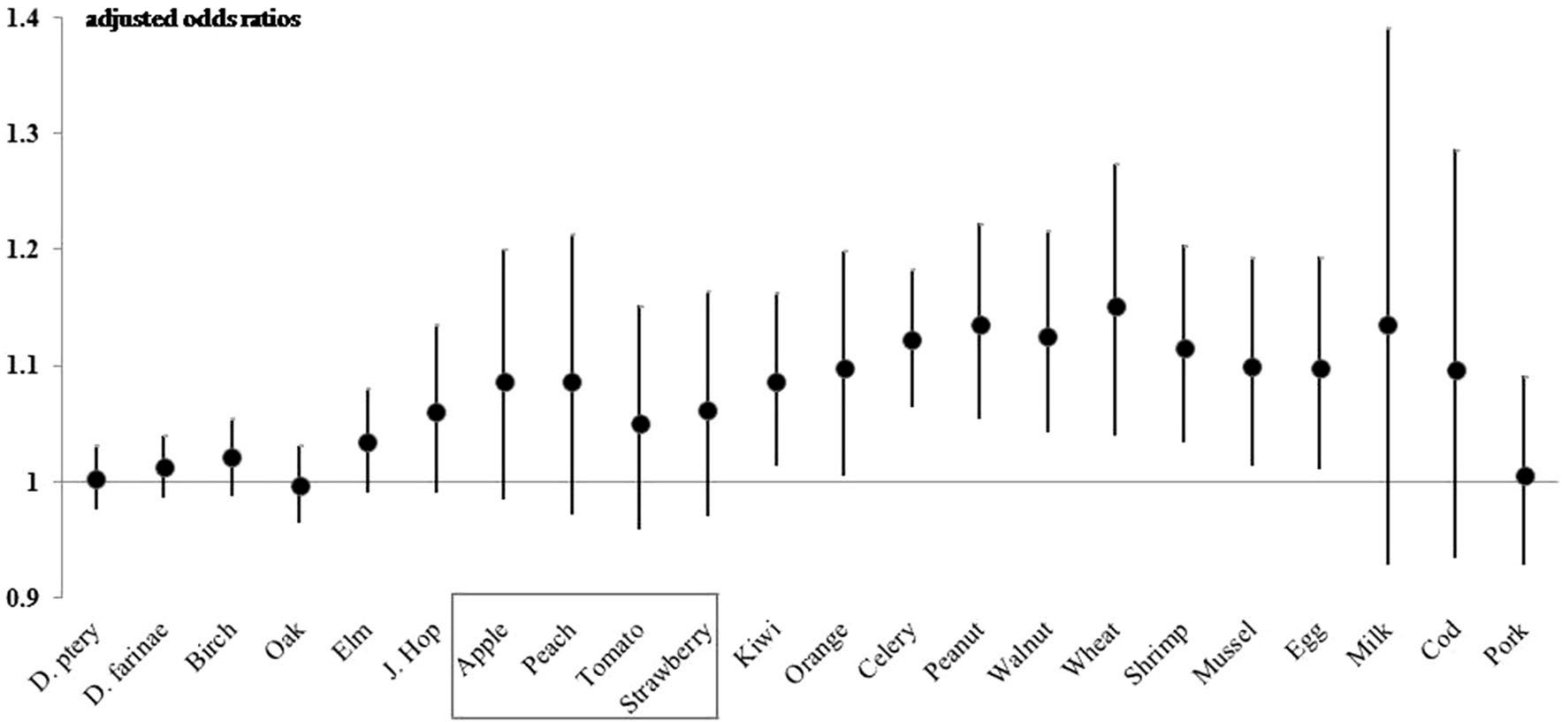
Adjusted odds ratios (with 95% confidence intervals) of the association of sensitization to individual allergens with serum LBP level. Adjusted for sex, age, body mass index z-score, parental asthma, allergic rhinitis, and atopic dermatitis, and household income.

We also analyzed the relationship of sensitization to individual allergens with LBP level (Fig. 2). Analysis of sensitization to plant allergens indicated that LBP level had significant associations with sensitization to kiwi (aOR 1.014, *P* = 0.018), orange (aOR 1.005, *P* = 0.039), celery (aOR 1.064, *P* < 0.001), peanut (aOR 1.054, 95% CI 1.135–1.223, *P* = 0.001), walnut (aOR 1.043, 95% CI 1.126–1.216, *P* = 0.003), and wheat (aOR 1.04, 95% CI 1.151–1.274, *P* = 0.007). LBP level was also significantly associated with sensitization to shrimp (aOR 1.034, 95% CI 1.115–1.204, *P* = 0.005), mussel (aOR 1.013, 95% CI 1.099–1.194, *P* = 0.024), and egg (aOR 1.011, *P* = 0.027). Partial correlation analyses between LBP levels and wheal diameter for each allergen in the skin prick test after adjustment for sex and age are shown in Suppl Table 3: Japanese hop, apple, peach, kiwi, orange, tomato, celery, peanut, walnut, wheat, egg, milk, mussel, and shrimp showed a significant positive correlation with LBP level.

## Discussion

The major finding of the current study is that the presence and severity of food allergic sensitization is positively associated with serum LBP level. This may suggest that LBP is associated with the active gut-derived inflammatory process that contributes to allergic sensitization. Of note, sensitization to celery, peanuts, nuts, and seafood were significantly associated with increased LBP level. This indicates that LBP may play a role in the development of sensitization to food allergens, and that serum LBP level may be useful as a surrogate biomarker for gut-inflammation. Sensitization to fruits may have had a relatively weak association with LBP level because their allergenic components are readily dissolved by gastric acid. To the best of our knowledge, this is the first study to report a positive association between LBP (i.e., a surrogate marker for gut-inflammation) and food allergic sensitization in human subjects.

It can be difficult to accurately measure serum LPS level. Only one previous study directly measured LPS and LBP levels in humans, and the results indicated that LBP and LPS levels were strongly correlated with each other^10^. Other studies have determined that serum LBP level can function as a biomarker for inflammation of the gut^4,9,22–24^ and the lung^25^. Our findings suggest that increased LBP level are associated with food allergic sensitization which is in agreement with these studies.

This significant association of serum LBP concentration with food allergic sensitization suggests that there may be mechanistic relationships. One possibility is that different allergens bind to LPS with different affinities^26,27^. When the LPS component of an allergen enters the systemic circulation, it can bind with LBP^28^, leading to CD14 binding to effector cells^29^ and activation of these cells^30,31^. This immune reaction may ultimately lead to innate immune disturbances, a systemic low-grade inflammatory reaction, and allergic sensitization^14,21^. Furthermore, LBP markedly enhances the sensitivity of TLR-expressing cells to lipoproteins^32^, thus promoting the antigen-presenting process. Although house dust mites^33^ and pollen^34^ contain LPS, food allergens do not. Hence, based on our findings, it is unclear whether a food allergen itself is the initiating factor of LBP-related chronic low-grade inflammation. Another mechanism is that LBP, which can function as a lipid transfer protein^10,27^ due to its hydrophobic binding sites for lipid ligands^27^, may bind directly to allergen lipids^27^. This could lead to immunomodulation by ligands of LBP and lipids on the surface of allergens, and a TH_2_-enhanced process that predisposes subjects to allergic sensitization due to an interaction of the innate immune system with invariant natural killer T cells^27^. This distinct function of LBP may explain our finding of a connection between chronic low-grade gut inflammation and food allergic sensitization. It is unclear whether the increased LBP produced by human cells following gut-inflammation in response to LPS is responsible for food allergic sensitization, or whether ingestion of food allergens directly cause gut inflammation by changing the gut microenvironment and thereby increasing LBP levels. When subjects predisposed to allergic diseases experience contact with allergens, this may cause upstream alterations in the respiratory system, skin, and/or gut microbiota, and these changes could promote changes in permeability, or cause microbial translocation and systemic low-grade LPS-related inflammation, a precondition for allergic sensitization^17^. In contrast, there are previous studies suggesting that LPS activity may modulate mucosal tolerance by inducing allergen-specific IgG1 production and distinct effector CDD4 + T cells with a mixed regulatory/Th1 phenotype^35^. Regardless of the mechanism, chronic low-grade gut inflammation due to dysregulation of microbiota may lead to increased levels of LPS and LBP, and this can markedly enhance the sensitivity of TLR-expressing cells to lipoproteins, thus promoting the antigen-presenting process and inducing food allergic inflammation in the gut.

High-sensitivity C-reactive protein (hs-CRP) is significantly associated with allergic sensitization. However, because we found that LBP level is associated with food allergic sensitization, but not aeroallergen sensitization, we speculate that LBP may be upregulated by gut low-grade inflammation, but not by systemic low-grade inflammation. In addition, our finding that LBP was not associated with allergic diseases may explain why gut inflammation occurs in children with dysregulation of immune responses related to food allergy, rather than in those with symptomatic or asymptomatic allergic diseases^21^.

The main strength of the current study is its population-based design. Furthermore, our data were from comprehensive and standardized objective assessments conducted by skilled and experienced research pediatricians at each school. Nevertheless, our study also has a few limitations. The cross-sectional design prevented us from inferring causal relationships between LBP level and food allergic sensitization. Allergen-specific IgE tests would be a much more reliable test marker to strictly demonstrate atopic immune responses. Much to our regret, we were not able to perform serum allergen-specific IgE tests due to the limited amount of blood sample from each subject. Moreover, because only a small number of the children had asthma, it may be inappropriate to conclude that LBP is not significantly associated with allergic diseases. Furthermore, an alternative explanation might reverse the cause-effect relation and assert that allergic sensitization leads to alterations in the respiratory system, skin, and/or gut microbiota. Another limitation is that there may be potential bias due to unrevealed factors that may influence serum LBP levels and interfere with the association between LBP levels and skin prick tests. In addition, since this was a survey against general school children, allergic conditions relied on self-reports and diagnoses of food allergy or asthma were not confirmed using spirometry or oral food challenge tests. Although the questionnaires we used were structured and validated^36^, the current study lacks confirmed diagnosis utilizing objective diagnosing tools.

## Conclusion

We demonstrated serum LBP level is associated with allergic sensitization, especially to sensitization to food allergens, in children from a general pediatric population. These results suggest that low-grade gut inflammation due to LBP-associated disturbance of innate immunity may be a key phenotype of food allergic sensitization. Further prospective studies are warranted to confirm the role of LBP-associated dysfunction of innate immunity in allergic sensitization and allergic diseases.

## Methods

### Study design

This study was conducted as a part of a health screening program conducted on school children of Seongnam, Korea. Detailed information was provided elsewhere^37^. Children from 3 kindergartens and 6 elementary schools in Seongnam, South Korea during June and July 2015 were enrolled. In this survey, the structured questionnaires were distributed to the parents of participants for investigation of demographic characteristics and allergic conditions. Subjects who showed apparent infection signs and/or symptoms such as fever, cough, rhinorrhea, phlegm, vomiting, and/or diarrhea prior or at the time of enrollment were excluded. Among the remaining children, 51 children refused blood sampling and 11 refused skin testing at the study site. An additional 146 children were excluded because there were unsatisfactory amount of samples available. Thus, 356 children aged 5–15 years (mean 9.6 years, 95% CI 9.4–9.8 years) were assessed in the final analysis.

### Exposures and outcomes

Parents answered a modified Korean version of the ISAAC questionnaire to determine the presence of signs and/or symptoms and the diagnosis of allergic diseases^36,38,39^. We also examined clinical factors that could affect serum LBP level in the study population using a structured questionnaire. Allergic diseases were defined as the presence of relevant symptoms within the last 12 months^37^. Trained nurses recorded anthropometric measurements in all children. The BMI z-score was based on age- and sex-standardized measures of adiposity in children from World Health Organization (WHO) growth standards^37^.

### LBP

The serum concentration of LBP was measured using a commercially available enzyme-linked immunosorbent assay (ELISA) kit (Duoset, R&D Systems, Minneapolis, MN, USA) according to the manufacturer’s instructions and with all measurements in duplicate. Serum samples were diluted 1000-fold before this assay. No significant cross-reactivity or interference with LBP was observed. A450 values with a calibration line obtained with an LBP standard preparation were recorded using an Infinite 2000 Pro Multimode Plate Reader (Tecan, Vienna, VA, USA).

### Skin prick test (SPT)

We conducted SPTs to assess allergic sensitization to 6 common aeroallergens (2 dust mites [*Dermatophagoides (D.) farinae* and *D. pteronyssinus*]; 3 tree pollens [birch, oak, and elm] and Japanese hop;10plant foods (apple, peach, kiwi, orange, tomato, strawberry, celery, peanut, walnut, and wheat); and 6 type I food allergens (egg, milk, cod, pork, mussel, and shrimp) using extract and control solutions from Lofarma (Milan, Italy)^37^. A subject was considered “sensitized” if there were one or more positive reactions (wheal diameter > 3 mm) to an allergen.

All children were categorized as being nonsensitized, monosensitized (positive SPT for a single antigen or multiple cross-reactive antigens without other positive tests), or polysensitized (positive SPTs for antigens in different classes)^40^.

### Additional laboratory tests

Blood samples were used to measure total eosinophil count (TEC), and serum 25-hydroxyvitamin D (25[OH]D) level. TEC was measured using an automated analyzer (Phadia AB, Uppsala, Sweden) and serum 25[OH]D level using a LIASON chemiluminescence immunoassay analyzer (DiaSorin, Stillwater, Minnesota). The participants were categorized into two groups according to TEC (< 4% or ≥ 4%), and 3 groups according to serum 25(OH)D level (< 20.0 ng/mL, deficient; 20.0–29.9 ng/mL, insufficient;or ≥ 30.0 ng/mL, sufficient)^41^.

### Ethics statement

This study was approved by the Institutional Review Board of the CHA Bundang Medical Center (IRB No. 2015-05-075), which abides by the Helsinki Declaration on ethical principles for medical research. Written informed consent was obtained from parents or guardians and consent/assent from the children.

### Statistical analysis

The normality of the distribution of LBP level was tested using the Kolmogorov–Smirnov test, and results are presented as medians (interquartile ranges [IQRs]). The significance of differences between continuous variables was assessed using the Mann–Whitney *U* test, Student’s *t*-test, or ANOVA, as appropriate. Categorical variables are presented as absolute numbers and proportions. The significance of differences in proportions was assessed using the Chi-square test. Pearson’s or Spearman’s correlation analysis was used to determine the association between serum LBP level and various clinical measures. The Holm method was used to counteract the problem of multiple comparisons. We analyzed the partial correlation of LBP with factors obtained by the structured questionnaire among the subdirectory data comprising questionnaire responses. Variables were included in the model if their *P* values were less than 0.1 or if they were a priori confounders (sex, age, and BMI z-score) in the multivariable analysis. The partial correlation analysis indicated that household income, breastfeeding, floor of residency, and type of residency had significant correlations with LBP (*P* < 0.1for all; Supple Table 4).To identify factors independently and significantly correlated with allergic diseases and allergic sensitization, a generalized linear regression analysis with the logit function was conducted. The outcomes (i.e., self-reported allergic diseases and allergic sensitization) were determined after adjustment for sex, age, BMI z-score, parental asthma, allergic rhinitis, atopic dermatitis, household income, breastfeeding, floor of residency, and type of residency in each model. All statistical analyses were performed using IBM SPSS Statistics ver. 25.0 (IBM Co., Armonk, NY, USA). All statistical tests were two-sided, and a *P* value below 0.05 was considered significant.

## Supplementary Information

Supplementary Information 1.

Supplementary Information 2.

Supplementary Information 3.

Supplementary Information 4.

Supplementary Information 5.

## Data Availability

The datasets generated during and/or analysed during the current study are available from the corresponding author on reasonable request.
