# Supplementary material for: Association of serum lipopolysaccharide-binding protein level with sensitization to food allergens in children
Source: Sci Rep. 2021 Jan 25;11:2143. doi: 10.1038/s41598-020-79241-x (PMC7835372; doi:10.1038/s41598-020-79241-x)
Supplement: Supplementary file 1 — Supplementary Information 1. [file 41598_2020_79241_MOESM1_ESM.docx]

**Suppl Table 1.** Clinical and demographic characteristics of study subjects (N=356, mean age = 9.6 years).

| **Characteristic** | **Subjects, n** | **%** |
| --- | --- | --- |
| Sex |  |  |
| Male | 177 | 49.7 |
| Female | 179 | 50.3 |
| BMI |  |  |
| Obese (≥95 percentile) | 9 | 2.5 |
| Overweight (85-95 percentile) | 34 | 9.6 |
| Non-obese (<85 percentile) | 313 | 87.9 |
| Allergic disease |  |  |
| Asthma | 19 | 5.3 |
| Allergic rhinitis | 167 | 47.2 |
| Atopic dermatitis | 80 | 22.5 |
| 25-hydroxyvitamin D |  |  |
| Normal | 11 | 3.1 |
| Insufficiency | 172 | 48.3 |
| Deficiency | 173 | 48.6 |
| Total eosinophil count |  |  |
| <4% | 219 | 61.5 |
| ≥4% | 137 | 38.5 |
| Atopic sensitization |  |  |
| Nonsensitization | 107 | 30.1 |
| Monosensitization | 160 | 44.9 |
| Polysensitization | 89 | 25.0 |

BMI, body mass index.

All children were categorized as being nonsensitized, monosensitized (positive SPT for a single antigen or multiple cross-reactive antigens without other positive tests), or polysensitized (positive SPTs for antigens in different classes).

Allergic diseases, such as asthma, allergic rhinitis, and atopic dermatitis, were defined as the presence of relevant symptoms within the last 12 months
