# Supplementary material for: Association of serum lipopolysaccharide-binding protein level with sensitization to food allergens in children
Source: Sci Rep. 2021 Jan 25;11:2143. doi: 10.1038/s41598-020-79241-x (PMC7835372; doi:10.1038/s41598-020-79241-x)
Supplement: Supplementary file 2 — Supplementary Information 2. [file 41598_2020_79241_MOESM2_ESM.docx]

**Suppl Table 2.** Association of serum LBP level with selected blood analytes among children (n = 356).

| **Hematologic factor** | **LBP (ng/mL)** | | | | |
| --- | --- | --- | --- | --- | --- |
|  | **Crude β (SE)** | ***P* value** |  | **Adjusted β (SE)** | ***P* value^*^** |
| 25-hydrovitamin D_3_ (ng/mL) | -0.003  (-0.011 to 0.005) | 0.429 |  | -0.002  (-0.011 to 0.007) | 0.617 |
| Hemoglobin, g/L | 0.000  (-0.052 to 0.051) | 0.986 |  | -0.014  (-0.070 to 0.042) | 0.621 |
| White blood cell count, ×10^9^/L | 0.015  (-0.015 to 0.044) | 0.338 |  | 0.010  (-0.020 to 0.040) | 0.499 |
| Neutrophils, ×10^9^/L | -0.001  (-0.006 to 0.003) | 0.561 |  | -0.003  (-0.008 to 0.002) | 0.295 |
| Eosinophils, % | -0.009  (-0.023 to 0.006) | 0.239 |  | -0.010  (-0.025 to 0.005) | 0.213 |

LBP, lipopolysaccharide-binding protein.

*^*^P* values are from linear regression after adjusting for age, sex, household income, and BMI z-score.

Each estimate is the β-coefficient (95% CI).
