# Supplementary material for: Association of serum lipopolysaccharide-binding protein level with sensitization to food allergens in children
Source: Sci Rep. 2021 Jan 25;11:2143. doi: 10.1038/s41598-020-79241-x (PMC7835372; doi:10.1038/s41598-020-79241-x)
Supplement: Supplementary file 3 — Supplementary Information 3. [file 41598_2020_79241_MOESM3_ESM.docx]

**Suppl Table 3.** Partial correlation analyses between LBP levels and wheal diameter for each allergen in the skin prick test after adjustment for sex and age.

|  | LBP | | |
| --- | --- | --- | --- |
|  | Rho | | *P* value |
| Aeroallergen |  |  | |
| *Dermatophagoides farinae* | 0.103 | 0.054 | |
| *Dermatophagoides pteronyssinus* | 0.087 | 0.102 | |
| Birch | 0.088 | 0.097 | |
| Oak | 0.021 | 0.700 | |
| Elm | 0.099 | 0.062 | |
| Japanese hop | 0.143 | **0.007** | |
| Plant food allergen |  |  | |
| Apple | 0.167 | **0.002** | |
| Peach | 0.186 | **<0.001** | |
| Kiwi | 0.157 | **0.003** | |
| Orange | 0.178 | **0.001** | |
| Tomato | 0.136 | **0.010** | |
| Strawberry | 0.094 | 0.077 | |
| Celery | 0.287 | **<0.001** | |
| Peanut | 0.260 | **<0.001** | |
| Walnut | 0.273 | **<0.001** | |
| Wheat | 0.215 | **<0.001** | |
| Type I food allergen |  |  | |
| Egg | 0.141 | **0.008** | |
| Milk | 0.125 | **0.018** | |
| Cod | 0.066 | 0.220 | |
| Pork | 0.003 | 0.954 | |
| Mussel | 0.149 | **0.005** | |
| Shrimp | 0.224 | **<0.001** | |
| Histamine | 0.030 | 0.575 | |
| Normal saline | 0.051 | 0.342 | |

LBP, lipopolysaccharide-binding protein.
