# Supplementary material for: Association of serum lipopolysaccharide-binding protein level with sensitization to food allergens in children
Source: Sci Rep. 2021 Jan 25;11:2143. doi: 10.1038/s41598-020-79241-x (PMC7835372; doi:10.1038/s41598-020-79241-x)
Supplement: Supplementary file 4 — Supplementary Information 4. [file 41598_2020_79241_MOESM4_ESM.docx]

**Suppl Table 1.** Identification of continuous and binary factors that could potentially confound analysis of serum LBP level.

|  | | LBP | | | | |  |
| --- | --- | --- | --- | --- | --- | --- | --- |
| Continuous variable^*^ | | B (95% CI) | *P* value |  | Adjusted^‡^ B (95% CI) | *P* value |  |
| Age | | -0.003 (-0.025 to 0.018) | 0.770 |  | -0.001 (-0.025 to 0.022) | 0.999 |  |
| BMI *z* score | | 0.037 (-0.004 to 0.078) | 0.079 |  | 0.036 (-0.005 to 0.078) | 0.089 |  |
| Binary variables | | Median (IQR) | *P* value ^†^ |  | Adjusted^‡^ B (95% CI) | *P* value |  |
| Sex^§^ | Male | 24.1 (17.1-30.0) | 0.927 |  | Ref | 0.929 |  |
|  | Female | 23.9 (17.8-29.9) |  |  | 0.996 (0.917 to 1.083) |  |  |
| House income^∥^ | Low | 22.0 (16.2-27.4) | **0.011** |  | Ref | **0.009** |  |
|  | High | 17.6 (30.7) |  |  | 1.121 (1.028 to 1.222) |  |  |
| Prematurity and/or LBW^¶^ | Yes | 25.6 (17.0-28.6) | 0.984 |  | Ref | 0.786 |  |
|  | No | 23.9 (17.5-30.0) |  |  | 1.020 (0.885 to 1.176) |  |  |
| Breastfeeding^**^ | No | 23.1 (15.7-28.5) | 0.124 |  | Ref | 0.082 |  |
|  | Yes | 24.5 (19.7-30.1) |  |  | 0.085 (-0.011 to 0.180) |  |  |
| Type of residency^††^ | Apartment | 25.5 (19.7-30.7) | **<0.001** |  | Ref | **<0.001** |  |
|  | Others | 21.6 (15.1-27.2) |  |  | 0.150 (0.069 to 0.231) |  |  |
| Floor of residency^‡‡^ | Basement/semi-basement first floor | 23.1 (15.7-28.5) | **0.003** |  | Ref | **0.002** |  |
|  | Second floor or higher | 24.5 (19.7-30.1) |  |  | 0.085 (-0.011 to 0.180) |  |  |

BMI, body mass index; IQR, interquartile range; LBP, lipopolysaccharide-binding protein.

^*^*P* values are from a generalized linear regression with the gamma function.

^†^*P* values are from non-parametric testing.

^‡^Adjusted for age, sex, and BMI z-score.

^§^There were 176 boys and 178 girls.

^∥^Low income was defined as less than 5,000,000 won (USD$ 4443.9) per month, and high income as greater than 5,000,000 won(USD $ 4443.9) per month.

^¶^For prematurity and/or LBW, 32 answered “yes” and 322 answered “no”. Prematurity was defined as a gestational age less than 37 weeks, and LBW as less than 2.5 kg.

^**^For feeding types, 272 answered breastfeeding and 77 answered formula-feeding.

^††^For type of residency, 225 children lived in apartments and 127 lived in residences other than apartments.

^‡‡^For floor of residency, 93 children lived in basement/semi-basement or first floor and 261 children lived in second floor or higher.

Numbers in bold indicate significant differences (*P*< 0.05).

The questionnaire collected information about *(i)* potential confounding factors (sex, age, height, weight, and body mass index [BMI] z-score); *(ii)* factors potentially associated with LBP (birth history, gestational age, birth weight, number of family members living together, birth order, mode of delivery, attendance at a daycare center, parental education, and socioeconomic status); *(iii)* eating habits during infancy (duration of breastfeeding and age at weaning); *(iv)* environmental factors (passive smoking at home, age of residence, floor and type of residence, presence of mold in the residence, presence of a fabric couch in the residence, type of ventilation in the residence window, keeping a pet at home, home renovation within 12 months, new furniture within 12 months, change of residence since birth, moving within 12 months, distance of the residence from a major road, and distance of the residence from a major road during pregnancy and birth); and *(v)* exercise.
