# Supplementary figures and images for: Association of serum lipopolysaccharide-binding protein level with sensitization to food allergens in children
Source: Sci Rep. 2021 Jan 25;11:2143. doi: 10.1038/s41598-020-79241-x (PMC7835372; doi:10.1038/s41598-020-79241-x)

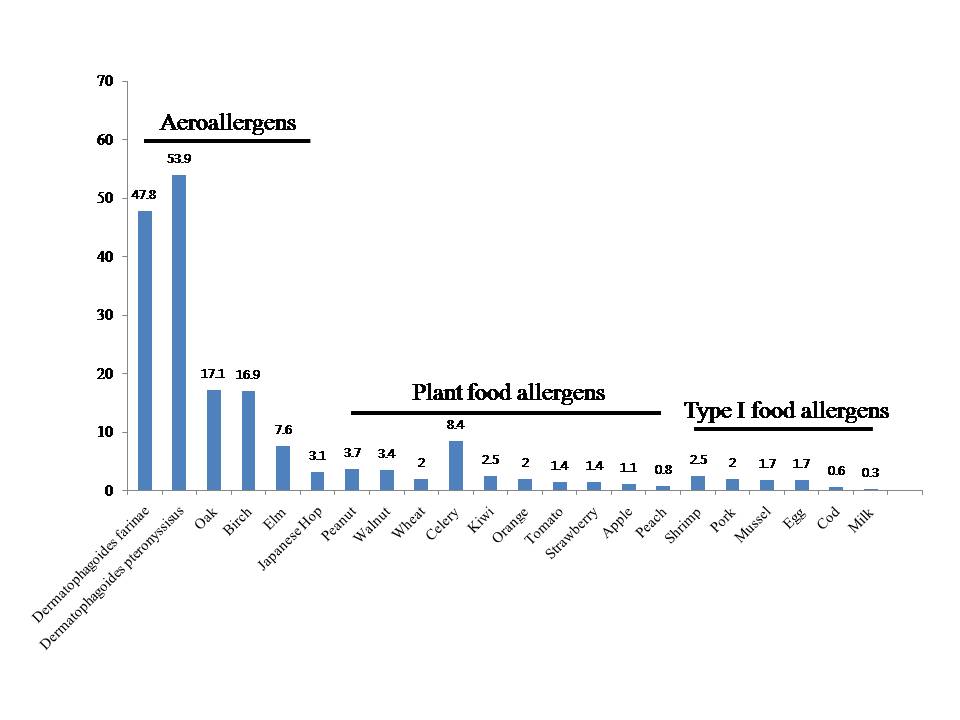

Supplement: Supplementary file 5 — Supplementary Information 5. [file 41598_2020_79241_MOESM5_ESM.jpg]
